# Unilateral Corneal Ectasia After Bilateral Transepithelial Photorefractive Keratectomy

**DOI:** 10.7759/cureus.76189

**Published:** 2024-12-22

**Authors:** Mo'ath AlShawabkeh, Ruaa Al Sakka Amini, Ayat Alni'mat, Muawyah D Al Bdour

**Affiliations:** 1 Department of Ophthalmology, The Hashemite University, Amman, JOR; 2 Family Medicine, Abdali Hospital, Amman, JOR; 3 Department of Ophthalmology, Henry Ford Health System, Detroit, USA; 4 Department of Ophthalmology, The University of Jordan, Amman, JOR

**Keywords:** cornea, ectasia, laser refractive surgeries, myopia, trans prk

## Abstract

We present the case of a 23-year-old male who experienced vision loss in his left eye 15 months after undergoing bilateral transepithelial photorefractive keratectomy (T-PRK). Despite the absence of any significant preoperative topographical risk factors in either eye, corneal ectasia was later confirmed in the left eye, while the right eye remained normal. Subtle asymmetry in topometric indices and a borderline high Index of vertical asymmetry (IVA) reading suggested the possibility of early subclinical keratoconus, potentially increasing the risk of post-refractive ectasia. The patient received corneal cross-linking (CXL) treatment in the affected eye to halt further progression, while the right eye remained under observation. This report reviews the rare instances of post-refractive ectasia. It highlights the potential role of subtle corneal irregularities in predisposing to ectasia, even without traditional risk factors.

## Introduction

Transepithelial photorefractive keratectomy (T-PRK) is a new emerging vision correction procedure designed to enhance traditional photorefractive keratectomy (PRK) by eliminating the need for mechanical removal of the corneal epithelium. This "no-touch" technique uses an excimer laser to remove the corneal epithelial layer and reshape the underlying stroma [1]. It has proven to be a safe and effective option for correcting low to moderate refractive errors, as serious postoperative complications are relatively low [2]. Additionally, T-PRK has shown advantages over PRK in terms of faster epithelial healing, reduced postoperative pain, and quicker visual recovery [3]. Post-refractive ectasia is a rare but serious complication that can occur after kerato-refractive surgeries. In ectasia, the cornea becomes progressively thinner and weaker, bulging outward and distorting vision. This condition resembles keratoconus, a relatively prevalent eye disorder characterized by progressive cornea thinning, changing its shape from dome to cone-like disease. Post-refractive ectasia can cause significant visual impairment, as it can result in increased myopia, irregular astigmatism, and reduced vision quality [4]. In this case report, we present a case of unilateral post-T-PRK ectasia in a patient with no apparent risk factors in both eyes.

## Case presentation

The patient initially presented in September 2021, seeking refractive surgery. Refraction results are shown in Table 1.

**Table 1:** Manifest refraction on initial presentation OD: Oculus dexter, OS: Oculus sinister, BCVA: Best-corrected visual acuity, MAR: Minimum angle of resolution.

Eye	Sphere	Cylinder	Axis	BCVA
OD	-3.00	-0.75	10	LogMAR 0.0
OS	-3.00	-0.50	10	LogMAR 0.0

**Figure 1:**
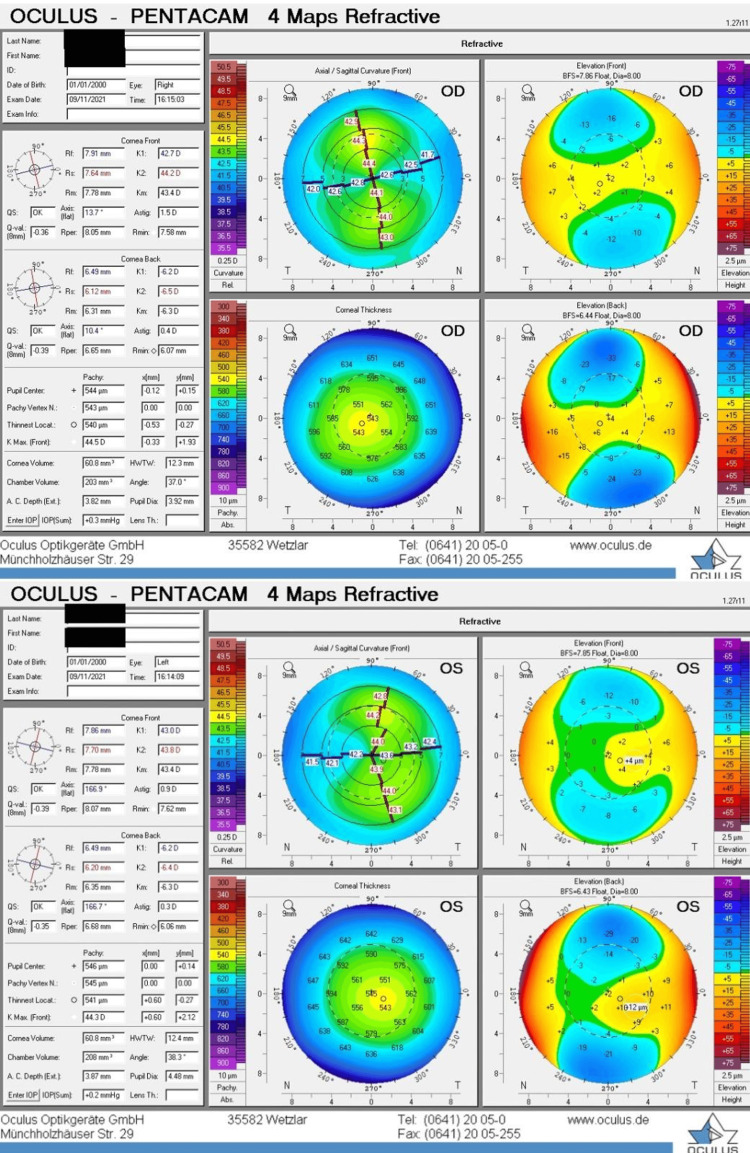
Preoperative corneal tomography map Preoperative corneal tomography for the right eye (above) and left eye (below): both eyes appear symmetrically normal and no risk factors for ectasia were seen bilaterally. OD: Oculus dexter, OS: Oculus sinister.

Pre-operatively, a corneal tomographic map was obtained. The right eye's maximum corneal curvature (Kmax) measured 44.5 D, with a corneal thickness of 540 μm at its thinnest point. In the left eye, Kmax was 44.3 D, and the thinnest corneal point measured 541 μm (Figure 1).

**Figure 2:**
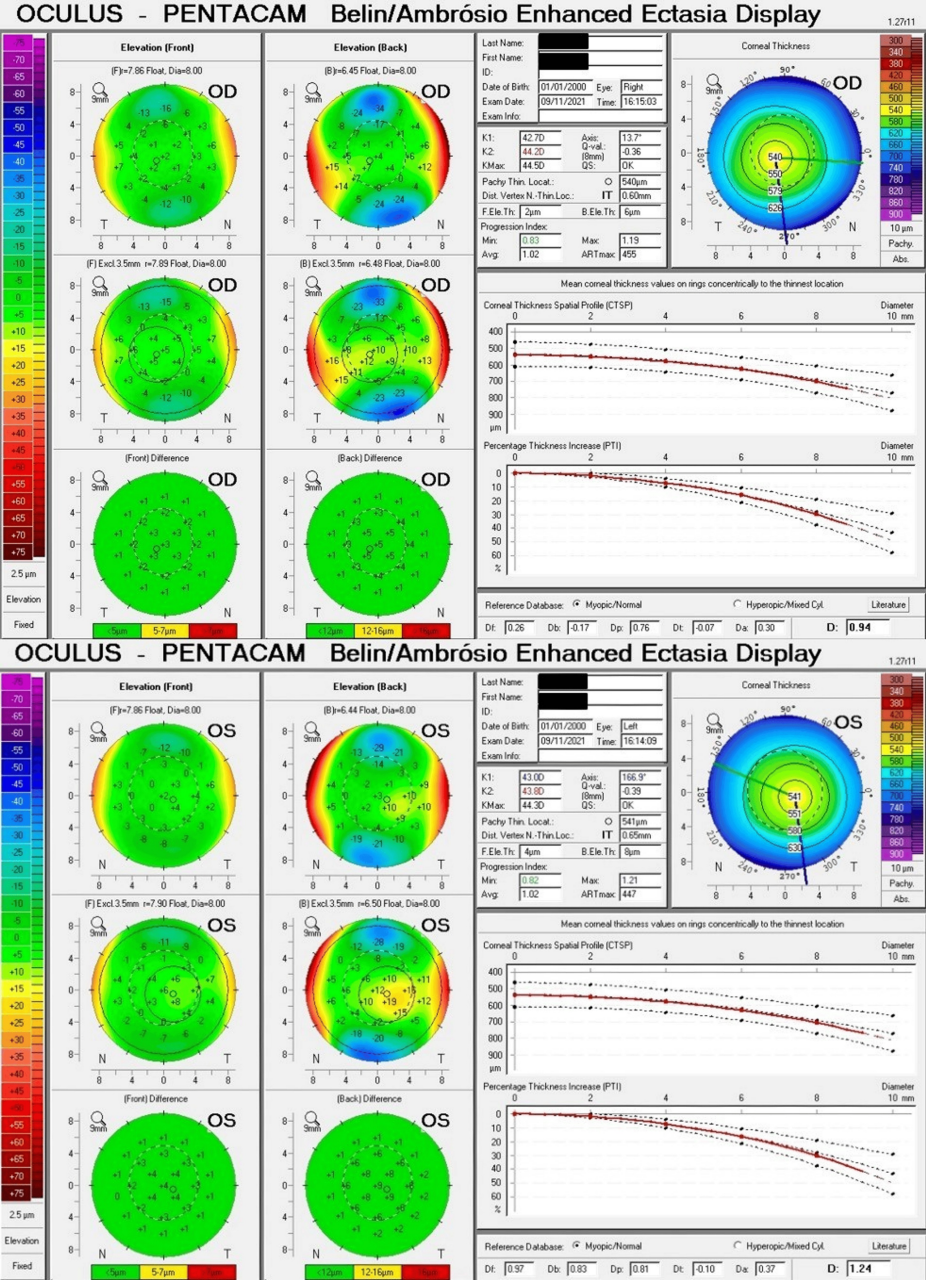
Belin/Ambrósio enhanced ectasia display preoperatively No high-risk features were noted. OD: Oculus dexter, OS: Oculus sinister.

No high-risk indices were evident in either cornea, and there was no obvious abnormality in the anterior or posterior elevation of his right and left corneas. Belin/Ambrósio Enhanced Ectasia Display (BAD_D value) was 0.94 and 1.24 in his right and left eye, respectively (Figure 2).

The patient underwent TranPRK in September 2021. The optical zone diameter was 6.7 mm in both eyes. The ablation depth (including the epithelium) was 116.5 microns in the right eye, with a residual stromal bed (RSB) thickness of 409 microns. In the left eye, the ablation depth was 112.4 microns, with an RSB thickness of 414 microns. There were no intraoperative complications. Following the procedure, the patient attended regular check-ups, during which his vision recovered well.

However, 15 months after the procedure, in January 2023, the patient began to experience a gradual decline in vision in his left eye, which could not be corrected with optical measures. His uncorrected visual acuity (UCVA) was LogMAR 0.0 (1.0) in the right eye and LogMAR 0.4 (0.4) in the left eye, where MAR is the minimum angle of resolution. The refraction results are shown here in Table 2.

**Table 2:** Manifest refraction 15 months postoperatively OD: Oculus dexter, OS: Oculus sinister, BCVA: Best-corrected visual acuity; MAR: Minimum angle of resolution.

Eye	Sphere	Cylinder	Axis	BCVA
OD	Plano	Plano	-	LogMAR 0.0
OS	-1.00	-1.50	65	LogMAR 0.40

**Figure 3:**
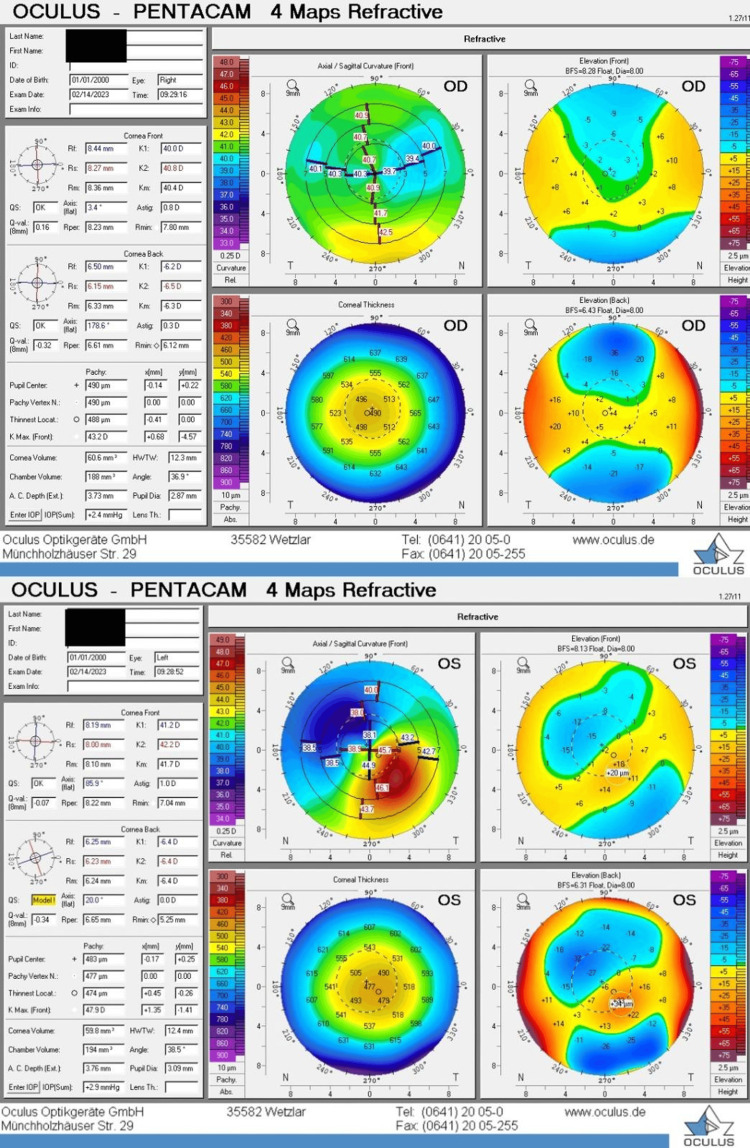
Postoperative corneal topography map Postoperative corneal topography for the right eye (above) and left eye (below): signs of corneal ectasia noted in the left eye, including inferior focal steepening on axial curvature map, a marked increase in keratometry readings, high Inferior-Superior (I-S) Asymmetry Index and increased elevation of the posterior corneal surface. None of these signs were seen in the right eye. OD: Oculus dexter, OS: Oculus sinister.

The anterior segment examination, optic nerve function, and fundus evaluation were unremarkable. The patient was prescribed lubricant eye drops, and a follow-up appointment for two weeks was scheduled for further assessment. Corneal topography results are presented in Figure 3.

Two weeks later, the uncorrected visual acuity remained LogMAR 0.0 (1.0) in the right eye but decreased to MAR 0.48 (0.3) in the left. The subjective and cycloplegic refraction results are illustrated in Tables 3, 4, respectively.

**Table 3:** Manifest refraction two weeks later OD: Oculus dexter, OS: Oculus sinister, BCVA: Best-corrected visual acuity, MAR: Minimum angle of resolution.

Eye	Sphere	Cylinder	Axis	BCVA
OD	Plano	Plano	-	LogMAR0.0
OS	-1.00	-2.00	100	LogMAR0.48

**Table 4:** Cycloplegic refraction OD: Oculus dexter, OS: Oculus sinister.

Eye	Sphere	Cylinder	Axis
OD	+1.00	-0.25	145
OS	+0.50	-1.50	86

Consequently, the patient was scheduled for monthly observations, during which topographical measurements and refractive errors were stable. Ultimately, the diagnosis of left post-refractive ectasia was made, for which he underwent corneal collagen cross-linking (CXL) in May 2023.

## Discussion

Corneal ectasia is a well-known complication of corneal laser refractive surgeries, which can result in severe visual impairment. It is characterized by an acquired, non-inflammatory, biomechanical outward bulging of the cornea in a keratoconus-like configuration. This condition leads to progressive corneal thinning with steepening centrally and inferiorly. Typically, it manifests as a myopic shift in refraction, increased regular and irregular astigmatism, and eventually a loss of best-corrected visual acuity (BCVA) [4]. Ectasia develops when refractive surgery, particularly laser in-situ keratomileusis (LASIK), compromises the biomechanic integrity of the cornea, resulting in a reduction of the load-bearing capacity of the anterior cornea, thereby increasing stress on the posterior corneal stroma which is less able to support the cornea’s structural demands. Factors placing the patient at increased risk for this condition include the preoperative thin cornea, high refractive error, young age, history of keratoconus, abnormal or irregular topography as well as topographical asymmetry between both eyes [5]. Also, the role of corneal biomechanical properties, such as viscoelastic changes and structural integrity, in addition to potential metabolic contributors, such as oxidative stress or alterations in keratocyte function, may predispose the cornea to ectatic progression.

According to Moshirfar et al. [6], post-refractive ectasia has been observed in eyes without preoperative identifiable risk factors with the following incidence rates: 20 per 100,000 eyes for PRK, 90 per 100,000 eyes for LASIK, and 10 per 100,000 eyes for small incision lenticule extraction (SMILE). The incidence of ectasia in LASIK was found to be 4.5 times higher than in PRK [7]. Validating the ectasia risk scoring system, Randlemann et al. found that an abnormal preoperative topographic pattern was the most significant factor associated with post-LASIK ectasia. Other contributing factors include younger age, thinner preoperative corneas, thinner residual stromal beds, high myopia, and a higher percentage of tissue altered [8]. When calculating the ectasia risk score for this case, the score was 3 for both eyes which is associated with low to moderate risk bilaterally.

The potential role of metabolic changes in affecting corneal biomechanical stability has been highlighted in a case report by Roszkowska et al. on a 54-years-old patient who developed post-PRK ectasia after 20 years of stable and successful PRK surgery. In their case, corneal ectasia developed following a bariatric surgery and a significant weight loss [9]. In our case, none of these risk factors were apparent at the time of T-PRK. Risk factors were excluded by examination and corneal topography. The patient was 21 years old at the time of the surgery and had a history of stable refraction, consistent weight, and stable metabolic condition.

In retrospect, the pre-operative topography revealed asymmetrical indices readings. The Vertical Asymmetry (IVA) index was notably different between the two eyes, measuring 0.04 in the right eye and 0.14 in the left eye, indicating a 0.10 difference between them. Additionally, the Index of Height Decentration was 0.003 on the right and 0.017 on the left. However, the Index of Surface Variance (ISV) was nearly symmetrical, with values of 16 and 17 in the right and left eyes, respectively.

The preoperative topographic readings of the left eye may suggest the presence of subclinical keratoconus. The Belin/Ambrosio Deviation Display (BAD_D), IVA, ISV, Index of height decentration (IHD), and 5th-order vertical coma aberration are recognized as key diagnostic criteria for subclinical keratoconus, with a correlation of R² = 0.65 and p < 0.001. Specifically, an IVA of 0.14 or higher is considered a cut-off for detecting subclinical keratoconus, which was observed in our case [10]. The importance of front surface curvature-derived indices (ISV, IVA, IHD) as a valuable parameter to detect keratoconus is highlighted in the literature; however, they can be normal in mild ectasia without front surface changes [11].

In this case, the ISV, IHD, and IVA were within normal, which suggests that it's not just the mere value that matters; it's also the asymmetry between the two eyes. Therefore, the most intriguing discovery here is the evident asymmetry between the topometric readings of the two eyes, which may be an important factor to consider when utilizing the suggested aberration indices to detect keratoconus suspects.

In recent years, deep learning (DL)-based artificial intelligence models have evolved as a promising tool for aiding the diagnosis of subclinical keratoconus and in the prevention of corneal ectasia by screening and identifying patients at risk of this complication [12]. Leveraging machine learning algorithms, these models can detect subtle patterns and interactions between multiple risk factors that may not be apparent through standard evaluations. Tools such as the Pentacam Random Forest Index (PRFI) and the Topographic and Biomechanical Index (TBI) synthesize data from corneal imaging into a single predictive score, making it easier for clinicians to assess the likelihood of ectasia with high precision [13,14]. With these predictive capabilities, AI-based models significantly improve patient safety by identifying high-risk candidates before surgery, thus reducing the incidence of postoperative complications like ectasia and enhancing long-term outcomes for refractive surgery patients.

## Conclusions

In conclusion, despite the absence of any known predisposing factors, unilateral post-T-PRK ectasia was confirmed in our case. The only notable findings were the apparent asymmetry in the topometric indices and a borderline high IVA reading in the left eye, suggesting the presence of subclinical keratoconus prior to the surgery. This asymmetry between the two eyes may be associated with an increased likelihood of post-refractive ectasia. Although the significance of this asymmetry remains to be elucidated in future studies, it is recommended to monitor patients exhibiting similar topographic asymmetries through regular follow-up visits. Enhanced imaging techniques and biomechanical assessments should be employed to track changes in corneal structure over time. Additionally, establishing a proactive management plan, which may include cross-linking or refraining from corneal laser refractive surgery in high-risk individuals, could mitigate the potential for ectasia. By integrating these monitoring strategies into clinical practice, practitioners can improve patient outcomes and reduce the incidence of postoperative complications.
